# Heteroscedastic Ridge Regression Approaches for Genome-Wide Prediction With a Focus on Computational Efficiency and Accurate Effect Estimation

**DOI:** 10.1534/g3.113.010025

**Published:** 2014-01-21

**Authors:** Nina Hofheinz, Matthias Frisch

**Affiliations:** Institute of Agronomy and Plant Breeding II, Justus Liebig University, 35392 Giessen, Germany

**Keywords:** genome-wide prediction, ridge regression, heteroscedastic marker variances, linkage disequilibrium, plant breeding populations, GenPred, Shared data resources

## Abstract

Ridge regression with heteroscedastic marker variances provides an alternative to Bayesian genome-wide prediction methods. Our objectives were to suggest new methods to determine marker-specific shrinkage factors for heteroscedastic ridge regression and to investigate their properties with respect to computational efficiency and accuracy of estimated effects. We analyzed published data sets of maize, wheat, and sugar beet as well as simulated data with the new methods. Ridge regression with shrinkage factors that were proportional to single-marker analysis of variance estimates of variance components (*i.e.*, RRWA) was the fastest method. It required computation times of less than 1 sec for medium-sized data sets, which have dimensions that are common in plant breeding. A modification of the expectation-maximization algorithm that yields heteroscedastic marker variances (*i.e.*, RMLV) resulted in the most accurate marker effect estimates. It outperformed the homoscedastic ridge regression approach for best linear unbiased prediction in particular for situations with high marker density and strong linkage disequilibrium along the chromosomes, a situation that occurs often in plant breeding populations. We conclude that the RRWA and RMLV approaches provide alternatives to the commonly used Bayesian methods, in particular for applications in which computational feasibility or accuracy of effect estimates are important, such as detection or functional analysis of genes or planning crosses.

Best linear unbiased prediction (BLUP) and Bayesian approaches were suggested by Meuwissen *et al.* (2001) for predicting genotypic values with DNA markers. These genome-wide prediction (GWP) approaches have proven to be useful in plant breeding populations (*cf*. Crossa *et al.* 2010; Albrecht *et al.* 2011; Hofheinz *et al.* 2012). To overcome the problem of overparameterization triggered by more available marker data (p) than number of observations (n), shrinkage factors (ridge regression; BLUP) or variable selection (Bayesian approaches) can be used. Shrinkage factors can be constant for all markers or marker-specific with the use of homo- or heteroscedastic genetic variances.

Homoscedastic genetic variances at all markers in the linear model are regarded as a major shortcoming of the BLUP approach because many traits are assumed to be controlled by only a subset of the genes of an individual, not by all of them. This shortcoming motivated the development of Bayesian approaches that allow for heteroscedastic marker variances but at the expense of being computationally demanding (*cf*. Meuwissen *et al.* 2001, Shepherd *et al.* 2010, Kärkkäinen and Sillanpää 2012). To avoid the computational demands of Bayesian approaches, a linear model approach that uses heteroscedastic marker variances for data sets with more genotypes than markers was proposed by Piepho (2009). The generalized ridge regression (heteroscedastic effects model, or HEM) of Shen *et al.* (2013) also allows marker-specific shrinkage for overparameterized situations. These authors emphasized the need for computationally efficient GWP approaches with heteroscedastic marker variances.

The accuracy of the predicted genotypic values for GWP approaches with homoscedastic and heteroscedastic marker variances was compared, *e.g.*, for fruit traits in apple (Kumar *et al.* 2012), *Fusarium* head blight resistance in barley (Lorenz *et al.* 2012), 13 traits important in wheat breeding (Heffner *et al.* 2011), and for eight data sets in wheat, barley, *Arabidopsis*, and maize data sets (Heslot *et al.* 2012). The common conclusion was that in most instances the accuracy of predicting genotypic values was comparable for the investigated approaches. In particular, (1) none of the approaches was clearly superior under a broad range of applications; and (2) the BLUP approaches proved to provide good prediction accuracies, even for traits that are not supposed to follow closely the infinitesimal model of quantitative genetics, such as resistances. This finding was confirmed in a simulation study by Wimmer *et al.* (2013), who recommend the use of BLUP in plant breeding populations with large linkage disequilibrium (LD) extent, small sample sizes, and medium trait heritabilities.

The focus of the aforementioned studies was on the prediction of genotypic values of the individuals of a prediction set, and high prediction accuracies were observed when the individuals of the training and the prediction set were related (*cf*. Hofheinz *et al.* 2012). If training and prediction sets are a finite population of related individuals, then long chromosome stretches are expected to be in LD. In such populations, it is sufficient for a high prediction accuracy of genotypic values that the effects of chromosome stretches in LD are estimated with high accuracy. A high accuracy of estimating the effects of single markers is not necessary. Even if the estimated effects of single markers might be different for the different GWP approaches, the sum of the effects on a chromosome stretch in LD might be of similar size. This can be regarded as an explanation why different GWP approaches with homoscedastic and heteroscedastic variances result in a prediction of gentoypic values of similar accuracy.

The focus of this research lies on the accuracy of GWP approaches with respect to estimating the effects of single markers. This accuracy is important for the identification and functional analysis of genes, for the identification of target genes for marker-assisted gene introgression programs, and for the prediction of the performance of crosses. Predicting crosses, *i.e.*, estimating expectation and variance of the performance of a population derived from a cross of two parental genotypes, is an application of GWP in which plant breeders have high expectations, but no reports of successful implementations have been published. Predicting crosses builds on modeling the breaking up of existing LD and the recombination of favorable alleles originating from the two parents of a cross. Both the accurate localization of markers linked to the investigated trait and the accurate estimation of the effects via a GWP approach are of central importance for the success of such a prediction.

Our objectives were (1) to present novel heteroscedastic ridge regression approaches that improve existing approaches with respect to computational efficiency or accuracy of effect estimates and (2) to demonstrate their properties with computer simulations and with data sets of maize, wheat, and sugar beet.

## Methods

### Linear model

For estimating the genetic effects of *m* biallelic single-nucleotide polymorphism (SNP) markers, a linear model, as follows, can be used:

$$y=1\beta_{0}+Zu+e,$$ (1)

**y** is the vector of *N* phenotypic values, *β*_0_ is a fixed intercept, **Z** is the design matrix relating the marker data to genotypes, **u** is the vector of genetic effects, and **e** is the vector of residuals. The elements of **Z** are coded as linear regression on the number of one of the two alleles, *i.e.*, as 0,1,2. The genetic effects *u_l_* (*l* = 1…*m*) and the residuals are normally distributed with $u_{l}∼\text{N}\left(0,\sigma_{l}^{2}\right)$ and $e_{k}∼\text{N}\left(0,\sigma_{e}^{2}\right)$ (*k* = 0…*N*). Furthermore, cov(*u_i_*, *u_j_*) = 0 (*i* ≠ *j*) and cov(*e_k_*, *e_l_*) = 0 (*k* ≠ *l*).

In ridge regression, the genetic effects *u_l_* are predicted by solving the following mixed-model equations

$$\left(\begin{array}{cc} 1\prime 1 & 1\prime Z\\ Z^{\prime }1 & Z^{\prime }Z+\Lambda^{2} \end{array}\right)\left(\begin{array}{c} \beta_{0}\\ u \end{array}\right)=(\begin{array}{c} 1^{\prime }y\\ Z^{\prime }y \end{array}),$$ (2)

where **Λ** is a diagonal matrix that defines the amount of shrinkage. If its elements *λ_l_* (*l* = 1…*m*) are defined as $\lambda_{l}=\sigma_{e}^{2}/\sigma_{l}^{2}$ and $\sigma_{l}^{2}=\sigma_{k}^{2}$ for all *l*, *k* ∈ {1…*m*}, then the predictions *u_l_* are the BLUPs (*cf*. Piepho 2009). This approach uses typically variance components $\sigma_{g}^{2}$ and $\sigma_{e}^{2}$ estimated from the data set under investigation.

In an approximative approach, preliminary rule of thumb estimates of the heritability $h_{p}^{2}$ can be used to define $\lambda_{l}=\left(1/h_{\text{p}}^{2}-1\right)m$ (ridge regression employing preliminary estimates of the heritability (RIR), Hofheinz *et al.* 2012). In the following, we suggest approaches to determine marker-specific shrinkage parameters *λ_l_* for ridge regression.

### Shrinkage by single-marker variance component estimates

A moment estimator of the variance component for each marker can be obtained from a random single-factor analysis of variance (ANOVA) as follows:

$${\hat{\sigma }}_{l}^{2*}=\frac{{\text{MQM}}_{l}-{\text{MQE}}_{l}}{\frac{1}{2}\left(N-\sum_{i}n_{i}^{2}/N\right)}.$$ (3)

MQM*_l_* and MQE*_l_* are the mean squares due to the marker and the error in the ANOVA for the *l*-th marker, *N* is the total number of individuals, and *n_i_* (*i* = 1,2,3) are the numbers of individuals in the three marker classes.

The ${\hat{\sigma }}_{l}^{2*}$ are not independent and, therefore, they do not sum up to the genetic variance, which means that they cannot be used directly to determine the shrinkage factor. However, they can be used to partition the total genetic variance to the individual markers:

$${\hat{\sigma }}_{l}^{2}={\hat{\sigma }}_{g}^{2}\;\frac{{\hat{\sigma }}_{l}^{2*}}{\sum_{l^{\prime }=1}^{m}{\hat{\sigma }}_{l^{\prime }}^{2*}}.$$ (4)

Here, the proportion of the genetic variance that is assigned to a marker *l* is proportional to the contribution of the single-marker ANOVA variance component of marker *l* to the sum of the single marker variance components of all markers. This results in shrinkage factors

$$\lambda_{l}=\frac{{\hat{\sigma }}_{e}^{2}}{{\hat{\sigma }}_{g}^{2}}\;\frac{\sum_{l^{\prime }}{\hat{\sigma }}_{l}^{2*}}{{\hat{\sigma }}_{l^{\prime }}^{2*}}.$$ (5)

The approach used to determine the shrinkage factors in Equation 5 is abbreviated as RMLA (*i.e.*, estimation of the error and genetic variance components with restricted maximum likelihood and partitioning according to ANOVA variance components).

The estimation of the genetic and error variance components from the data set under consideration can be replaced by using preliminary estimates of the heritability $h_{p}^{2}$ as suggested by Hofheinz *et al.* (2012). This results in shrinkage factors

$$\lambda_{l}=\left(1/h_{\text{p}}^{2}-1\right)m\;\frac{\sum_{l^{\prime }}\sigma_{l}^{2*}}{\sigma_{l^{\prime }}^{2*}}.$$ (6)

We abbreviate this procedure RRWA (*i.e.*, ridge regression with weighing factors according to ANOVA variance components).

### Shrinkage by fixing the residual variance in variance component estimation

BLUPs of **u** in a linear model as defined by Equation 1 can be obtained with an iterative procedure on basis of the expectation-maximization algorithm (Searle *et al.* 1992) that consists of solving the mixed-model equations in Equation 2 for the parameter vector and then solving the following,

$$\begin{array}{l} {\hat{\sigma }}_{e}^{2}=\left(y^{\prime }y-{\hat{b}}^{\prime }X^{\prime }y-{\hat{u}}^{\prime }Z^{\prime }y\right)/\left(N-1\right)\\ {\hat{\sigma }}_{l}^{2}=\left({\hat{u}}_{l}^{\prime }{\hat{u}}_{l}-{\hat{\sigma }}_{e}^{2}\text{tr}C_{\mathrm{ll}}\right)/q_{l} \end{array}$$ (7)

for the variance components until convergence is reached (Misztal and Schaeffer 1986). Here *q_l_* is the number columns of the design matrix **Z** that correspond to the variance component $\sigma_{l}^{2}$ and tr**C***_ll_* is the trace of the inverse of the coefficient matrix of Equation 2 that corresponds to the variance component.

If $\sigma_{l}^{2}=\sigma_{k}^{2}$ (*l*, *k* ∈ {1…*m*}) is the constant variance of marker effects, **C***_ll_* is the complete coefficient matrix, and *q_l_* the number of columns of **Z** (assuming full column rank), then the procedure can be used to obtain the variance components that yield the BLUPs.

A modification can be used to determine marker-specific shrinkage factors for ridge regression. First, ${\hat{\sigma }}_{e}^{2}$ is estimated as with BLUP. Then, the iterative procedure is repeated, but with two modifications: (1) The residual error $\sigma_{e}^{2}$ is not updated in each iteration round but instead the residual variance is held fixed for the value estimated in the first round. (2) For each marker, a different ${\hat{\sigma }}_{l}^{2}$ is estimated. This results in *m* values for ${\hat{\sigma }}_{l}^{2}$ and those are used to define the shrinkage factor for ridge regression as $\lambda_{l}={\hat{\sigma }}_{e}^{2}/{\hat{\sigma }}_{l}^{2}$. We abbreviate this procedure RMLV (*i.e.*, modification of the restricted maximum likelihood procedure that yields heteroscedastic variances).

### Software

We implemented the RIR, RMLA, RRWA, and RMLV approaches in our software SelectionTools (www.uni-giessen.de/population-genetics/downloads), which was also used for computer simulations. To perform reparametrized BLUP we used the R package rrBlupMethod6 (Piepho *et al.* 2012). The package BLR (Pérez *et al.* 2010) was applied for performing the Bayesian LASSO (BL). We used 1500 iterations and discarded the first 500 iterations as burn-in. The R package bigRR (Shen *et al.* 2013) was used for the HEM approach. A summary of all approaches used in the present study is given in Table 1. The code for all calculations is available in the Supporting Information, File S1, File S2, File S3, and File S4.

**Table 1:** Summary of GWP approaches organized by the assumption of marker variances in the present study

Approach	Marker Variances		Reference/R Package
	Homoscedastic	Heteroscedastic	
BLUP	x		Meuwissen *et al.* (2001)
rrBlupM6	x		Piepho *et al.* (2012)
RIR	x		Hofheinz *et al.* (2012)
BL		x	Pérez *et al.* (2010)
HEM		x	Shen *et al.* (2013)
RMLA		x	New approach
RMLV		x	New approach
RRWA		x	New approach

GWP, genome-wide prediction; BLUP, best linear unbiased prediction; RIR, ridge regression employing preliminary estimates of the heritability; BL, Bayesian LASSO ; HEM, heteroscedastic effects model; RMLA, estimation of the error and genetic variance components with restricted maximum likelihood and partitioning according to analysis of variance components; RMLV, modification of the restricted maximum likelihood procedure that yields heteroscedastic variances; RRWA, ridge regression with weighing factors according to analysis of variance components.

### Experimental data sets

Three experimental data sets were used to investigate the prediction accuracy, size of effect estimates, and computing time of GWP approaches. The first data set consisted of 300 tropical maize lines from the International Maize and Wheat Improvement Center (CIMMYT), which were genotyped with 1148 SNP markers (Crossa *et al.* 2010). The traits grain yield (GY), female flowering, male flowering, and anthesis-silking interval were analyzed. Each trait was evaluated under severe drought stress and well-watered conditions.

The second data set consisted of 306 elite wheat lines from CIMMYT, which were genotyped with 1717 diversity array technology markers (Pérez-Rodríguez *et al.* 2012). The averages of all employed environments for the traits GY and days to heading were analyzed. The maize and the wheat data sets are available as an online supplement to the publications. The third data set consisted of 310 inbred lines from a commercial sugar beet breeding program, which were genotyped with 300 SNP markers (Hofheinz *et al.* 2012). The traits sugar content and molasses loss were analyzed. Genotypic and phenotypic data for both traits are available in the File S4.

To assess the accuracy of predicting genotypic values, we used repeated random subsampling to divide the data for cross validation. The first subset was used to estimate the marker effects and contained 80% of the data. The second subset contained 20% of the data and was used to validate the effects. The correlations between observed and predicted values were averaged over 100 cross validation runs.

### Simulations

Computer simulations were used to investigate prediction accuracy of GWP approaches with respect to map position and effect size. To investigate the effect of high and low LD, we simulated random intermating of a large F1 population for either three or 19 generations (ngen = 3, 19). From the last intermating generation, 600 random doubled haploid lines were developed. We simulated 10 chromosomes, each of 1.6 M length, which were evenly covered with markers. To investigate the effect of high, medium, and low marker density, we considered distances between two adjacent markers of 1 cM, 2 cM, or 5 cM (md = 1, 2, 5). Two genes affected the trait on each chromosome; they were 0.401 M and 1.201 M distant from the telomere. Each had a positive effect of 2.5 on the trait. Both favorable alleles originated from the same parental line of the F1 population. To obtain phenotypic values, for each of the 600 doubled haploid lines, a random normally distributed residual was added to the genotypic value. The residual effect was chosen such that the heritability of the trait was *h*^2^ = 0.5 or *h*^2^ = 0.8. Estimation of marker effects in the simulated data set was replicated 50 times for each GWP approach and the estimated effects sizes for each marker were averaged over the replications.

## Results

### Computational efficiency

The computing time required to estimate marker effects with the simulated and experimental data sets was compared with a Linux workstation with 8 GB RAM and an Intel Core Quad 2.80 GHz processor. Among the approaches with homoscedastic marker variances, RIR was the fastest, and among those with heteroscedastic marker variances, RRWA was the fastest (Table 2). With both approaches, marker effect estimation took less than a second for all investigated data sets. RMLV was the slowest approach; in particular, for large data sets, the required computing time was considerable greater than that required for the other approaches.

**Table 2:** Computing time (sec) required for the estimation of marker effects with different GWP approaches

	Homoscedastic Marker Variances			Heteroscedastic Marker Variances				
	RIR	BLUP	rrBLUPM6	RMLV	RRWA	RMLA	BL	HEM
Simulated data, 500 individuals								
330 markers	0.03	0.16	0.91	5.07	0.05	0.16	5.14	39.92
810 markers	0.05	3.18	1.55	50.30	0.13	3.38	7.99	49.56
1610 markers	0.23	32.11	1.68	330.60	0.30	28.22	11.77	63.65
Crossa *et al.* (2010), 264 maize lines								
1135 SNP markers	0.10	9.08	0.37	118.20	0.14	9.17	11.10	8.79
Pérez-Rodríguez *et al.* (2012), 306 wheat lines								
1717 DArT markers	0.23	61.8	0.62	405.60	0.37	60.60	8.96	12.49
Hofheinz *et al.* (2012), 310 sugar beet lines								
300 SNP markers	0.01	0.12	0.35	3.72	0.04	0.11	5.51	3.69

For the maize data set, the trait GY-WW was investigated, for the wheat data set the trait GY, and for the sugar beet data set the trait SC. GWP, genome-wide prediction; RIR, ridge regression employing preliminary estimates of the heritability; BLUP, best linear unbiased prediction; RMLV, modification of the restricted maximum likelihood procedure that yields heteroscedastic variances; RRWA, ridge regression with weighing factors according to analysis of variance components; RMLA, estimation of the error and genetic variance components with restricted maximum likelihood and partitioning according to analysis of variance components; BL, Bayesian LASSO; HEM, heteroscedastic effects model; SNP, single-nucleotide polymorphism; DArT, diversity array technology; GY, grain yield; WW, well-watered; SC, sugar content.

### Prediction accuracy of GWP approaches

For the approaches BLUP, RRWA, RMLA, BL, and HEM, the correlation between predicted and observed phenotypic values ranged between 0.31 for flowering time in the maize data set and 0.86 for molasses loss in the sugar beet data set (Table 3). The differences in prediction accuracy between the data sets were pronounced; however, a clear trend with respect to differences between the GWP approaches was not observable. Prediction accuracies were nearly identical for the approaches BLUP, RIR, and RRBlupM6; therefore, only the results for BLUP are presented. The RMLV approach showed considerable lower prediction accuracies than the other approaches, ranging from ***r*** = 0.19 to 0.82. Similar trends were observed with the simulated data (data not shown).

**Table 3:** Correlation between observed and predicted phenotypic values determined with cross validation for different traits in the maize, wheat, and sugar beet data sets

Trait-Environment		Heteroscedastic Marker Variances				
	BLUP	RMLV	RRWA $\left(h_{\text{p}}^{2}\right)$	RMLA	BL	HEM
Crossa *et al.* (2010), 284 maize lines (264 lines, GY)						
MFL-WW	0.36	0.28	0.35 (0.8)	0.38	0.36	0.35
MFL-SS	0.45	0.28	0.38 (0.8)	0.39	0.45	0.44
FFL-WW	0.31	0.27	0.32 (0.8)	0.31	0.31	0.32
FFL-SS	0.51	0.35	0.46 (0.8)	0.47	0.48	0.50
ASI-WW	0.51	0.35	0.50 (0.8)	0.52	0.51	0.47
ASI-SS	0.51	0.35	0.44 (0.8)	0.46	0.50	0.45
GY-WW	0.54	0.36	0.46 (0.9)	0.50	0.54	0.52
GY-SS	0.43	0.19	0.34 (0.9)	0.37	0.43	0.35
Pérez-Rodríguez *et al.* (2012), 306 wheat lines						
GY-average	0.65	0.54	0.66 (0.8)	0.66	0.63	0.63
DTH-average	0.59	0.41	0.57 (0.9)	0.60	0.58	0.55
Hofheinz *et al.* (2012), 310 sugar beet lines						
SC	0.83	0.78	0.80 (0.9)	0.80	0.83	0.82
ML	0.85	0.82	0.84 (0.4)	0.86	0.86	0.85

For the RRWA approach, the preliminary heritability estimates $h_{\text{p}}^{2}$ are given in brackets. BLUP, best linear unbiased prediction; RMLV, modification of the restricted maximum likelihood procedure that yields heteroscedastic variances; RRWA, ridge regression with weighing factors according to analysis of variance components; RMLA, estimation of the error and genetic variance components with restricted maximum likelihood and partitioning according to analysis of variance components; BL, Bayesian LASSO; HEM, heteroscedastic effects model; GY, grain yield; MFL, male flowering; WW, well-watered; SS, severe drought stress; FFL, female flowering; ASI, anthesis-silking interval; DTH, days to heading; SC, sugar content; ML, molasses loss.

### Size of effect estimates in the wheat data set

**Figure 1:**
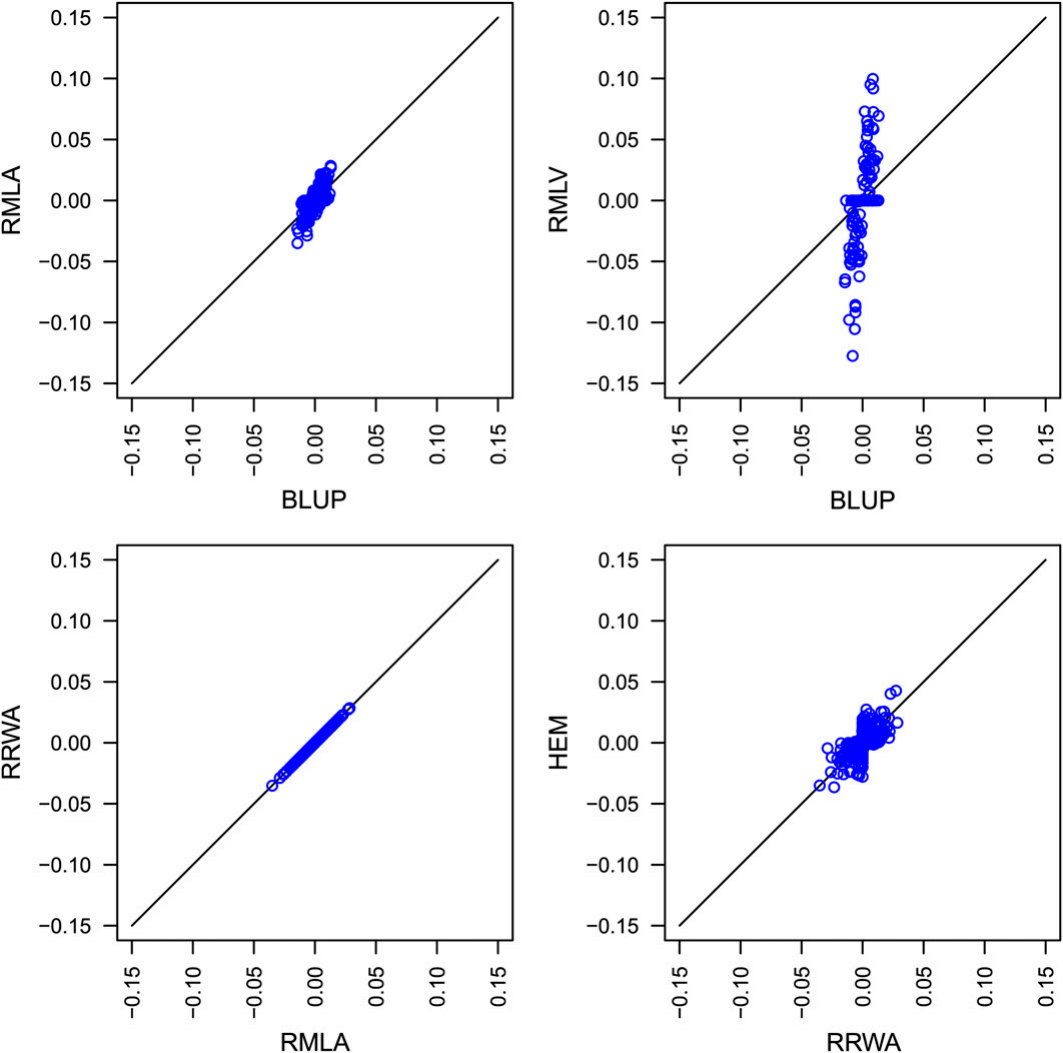
Comparison of the estimated marker effects for grain yield (GY) in the wheat data set for the best linear unbiased prediction (BLUP), ridge regression with weighing factors according to analysis of variance components (RRWA), estimation of the error and genetic variance components with restricted maximum likelihood and partitioning according to analysis of variance components (RMLA), modification of the restricted maximum likelihood procedure that yields heteroscedastic variances (RMLV), and heteroscedastic effects model (HEM) approaches.

In the wheat data set for the trait GY, markers for which the effects estimated with the BLUP approach were high had even greater effects with the RMLA approach (Figure 1). With RMLV, the differences in size between small and large effects were even greater. Most marker effects were shrunken to zero, and only a subset of markers had remarkably high effect estimates. The approaches RRWA and RMLA estimated marker effects of identical effect sizes. HEM and RRWA estimated marker effects of comparable magnitude. Both shrank many marker effects toward zero and estimated greater effects for the remaining markers. However, the marker effects shrunken near zero were not the same for both approaches.

### Simulation study on accuracy of marker effect estimates

**Figure 2:**
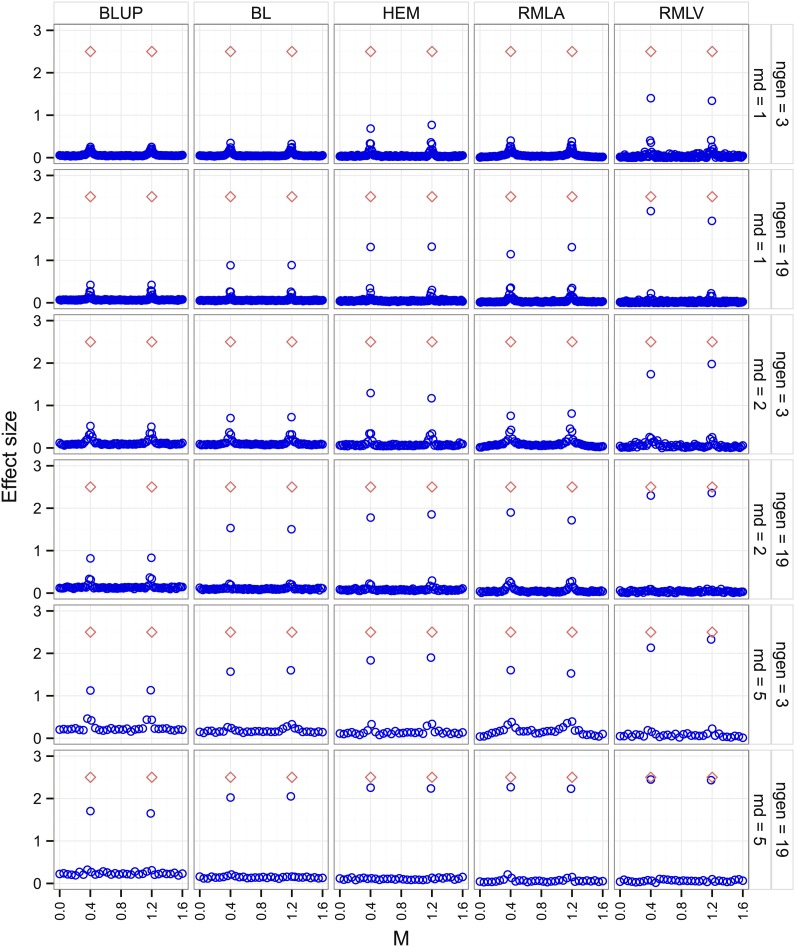
Marker effects (blue circles) estimated with different GWP approaches in the simulated data set plotted against marker locations [M] for the first chromosome. The positions of the simulated quantitative trait loci are symbolized by open red diamonds, ngen is the number of random intermating generations, and md is the marker distance [cM] of two adjacent markers.

For all combinations of marker distance (md = 1, 2, 5) and LD (ngen = 3, 19) the BLUP approach estimated the true marker effects with the least accuracy and the RMLV approach with the greatest accuracy (Figure 2). The BL, HEM, and RMLA approaches reached greater accuracies than the BLUP approach but still were outperformed considerably by the RMLV approach.

The accuracy of the BLUP approach was in particular low for the combination of small marker distances (md = 1) and high LD (ngen = 3). Here only RMLV resulted in usable effect estimates. With decreasing marker distances and decreasing LD the accuracy of the effect estimates obtained by the BLUP approach increased. However, the other approaches still provided effect estimates with considerable greater accuracy.

The greatest accuracy of effect estimates was achieved for large marker distances (md = 5) and low LD (ngen = 19), but still the BLUP showed a considerable underestimation of the true effects.

In addition to the simulations with a heritability of *h*^2^ = 0.8 (Figure 2), we performed the same set of simulations with a heritability of *h*^2^ = 0.5. The accuracy of effect estimates was lower but showed the same trends as with *h*^2^ = 0.8 (File S5).

## Discussion

### Heteroscedastic marker variances

For highly polygenic traits that follow closely the infinitesimal model of quantitative genetics, like yield, GWP approaches assuming homoscedastic marker variances are expected to be efficient for predicting genotypic values. However, GWP approaches with heteroscedastic marker variances model better the genetic basis of traits when the number of markers is substantially greater than number of genes underlying the trait. This is the case for SNP maps with high marker densities or for traits that are controlled by only a few genes. Bayesian models were the first heteroscedastic GWP approaches. Their two main drawbacks are that choosing a suitable prior is required and that they are computationally very demanding. Dense marker maps have become state of the art and aggravate the problem of high computing times required for Bayesian approaches. Hence, fast and efficient heteroscedastic GWP approaches are necessary (*cf*. Shen *et al.* 2013).

Our RMLA approach, as well as the HEM approach of Shen *et al.* (2013), provides computational efficient alternatives to Bayesian approaches. The core of both approaches is to determine an individual shrinkage factor for each marker and then apply these shrinkage factors in ridge regression. The shrinkage factors for HEM are determined on basis of a BLUP estimate of the marker effects *u_i_*, whereas RMLA uses a single-marker ANOVA. From a computational point of view, obtaining the BLUP estimates requires iterative procedures, whereas RMLA requires only the calculation of sums of squares. Consequently, determining shrinkage factors for RMLA is simpler and faster than for HEM. A second property that distinguishes RMLA from HEM is that the shrinkage factors for HEM are based on a first approximation, which uses homoscedastic marker variances; in contrast, the shrinkage factors for RMLA are based on a first approximation using heteroscedastic marker variances.

The computational efficiency of HEM was similar to that of RMLA for the data set of Crossa *et al.* (2010), but HEM was faster than RMLA for the data set of Pérez-Rodríguez *et al.* (2012) (Table 2). This advantage can be attributed to the optimized fitting algorithm of HEM, which makes its running time proportional to the number of individuals and not to the number of markers, as is the case for RMLA. Adopting a similar approach for RMLA might provide increased performance for dense marker maps. We chose to implement a different strategy for obtaining better performance. Approximating RMLA with RRWA uses preliminary estimates of the heritability instead of estimating the genetic and the residual variance from the data set under investigation. This results in a heteroscedastic ridge regression approach that does not need iterative procedures at all. RMLA and its approximation RRWA yielded the same effect estimates (Figure 1), and estimating the marker effects with RRWA took less than 1 sec for medium-sized data sets (Table 2). RRWA outperformed the other investigated approaches by factors between 10 and 100.

### Prediction of genotypic values and size of estimated effects

The accuracy of predicting genotypic values was comparable for homo- and heteroscedastic genetic GWP approaches (*cf*. Heffner *et al.* 2011). Our results confirm that in general no advantage of a particular approach can be observed with respect to predicting genotypic values (Table 3). The size of effect estimates, however, was clearly different in the wheat data set of Pérez-Rodríguez *et al.* (2012) for the five investigated GWP approaches (Figure 1). The estimated effects for grain yield were greater for RMLA and HEM than for BLUP. RMLV resulted in the greatest effects and the most effects shrunken near zero. Hence, the similarity of GWP approaches with respect to predicting genotypic values is not caused by similar estimated marker effects.

We conclude that a high accuracy of estimated marker effects is not a prerequisite for high prediction accuracies of genotypic values, as long as marker alleles that were in positive LD in the estimation set are still to a large extent in positive LD in the individuals for which the genetic values were predicted. However, because there are considerable differences in the estimated marker effects between the GWP approaches, the choice of the GWP approach is expected to have an impact on the success of such applications of GWP that rely on the accuracy of estimates of single marker effects.

### Importance of accurate effect estimates

Identification of known candidate genes in *Arabidopsis* (Shen *et al.* 2013) and apple (Kumar *et al.* 2012) was possible with effect estimates obtained by heteroscedastic GWP approaches. In contrast, no successful identification of genes was reported with results from homoscedastic BLUP estimates. This can be regarded as an indication that the greater effects obtained by the heteroscedastic approaches (Figure 1) are modeling the genetic basis of traits controlled by few genes better than homoscedastic BLUP and that accurate marker effect estimates are a prerequisite for the identification and fine mapping of functional genes.

An application of GWP that is most anticipated by plant breeders is planning crosses. In planning crosses, the probability distribution of the genotypic values of a population is investigated, which was derived from the cross of two parents with known phenotype and marker genotype. This distribution depends on the recombination between loci in the two parents of the cross, which breaks up the LD present in the parents. Here, it is not sufficient that the sum of genotypic values on a chromosome stretch in LD is correctly estimated. Instead, the effect of each single marker needs to be estimated with high accuracy. These two applications demonstrate that there is a need for GWP that provide accurate effect estimates for single markers.

With experimental data sets, the differences between GWP approaches with respect to effects sizes can be investigated (Figure 1), but it is not possible to evaluate which of the different effects at a marker is in fact the better estimate of the true (but unknown) effect. The importance of the two aforementioned applications and the fact that with experimental data the true effects are unknown motivated our simulation study.

### Accuracy of effect estimates depending on the GWP approach

In breeding populations of crop species, the level of LD is typically high. Li *et al.* (2011) observed 20.6 cM for sugar type inbreds of sugar beet, and Stich *et al.* (2005) found an average LD length of 33 cM in European elite maize germplasm. The simulations with high marker density (md = 1) and high LD (ngen = 3) represent such a genetic situation (Figure 2). Here BLUP estimates of the genetic effects of traits controlled by two genes are underestimated. The underestimation is so severe that a useful application of the BLUP effect estimates seems unrealistic. Although there is still a considerable underestimation of RMLV in this scenario, this approach was the only that provided an effect estimate useful for applications like prediction of crosses and identification of functional genes.

In conclusion, our results confirm the results of previous studies that the BLUP can provide accurate predictions of genotypic values. However, for dense markers and strong LD, the effect estimates of BLUP are very imprecise. For applications of GWP that rely on accurate effect estimations, heteroscedastic approaches are superior. In particular, the RMLV approach is a promising approach for providing accurate GWP effect estimates.

## Supplementary Material

Supporting Information
