# Supplementary material for: Heteroscedastic Ridge Regression Approaches for Genome-Wide Prediction With a Focus on Computational Efficiency and Accurate Effect Estimation
Source: G3 (Bethesda). 2014 Jan 21;4(3):539–46. doi: 10.1534/g3.113.010025 (PMC3962491; doi:10.1534/g3.113.010025)
Supplement: Supporting Information [file supp_4_3_539__index.html]

Heteroscedastic Ridge Regression Approaches for Genome-Wide Prediction With a Focus on Computational Efficiency and Accurate Effect Estimation — Supporting Information 

# Heteroscedastic Ridge Regression Approaches for Genome-Wide Prediction With a Focus on Computational Efficiency and Accurate Effect Estimation

## Supporting Information for Hofheinz and Frisch, 2014

**Files in this Data Supplement:**

- File S1 - Genotypic and phenotypic data of Pérez-Rodríguez et al. (2012), recoded; R code for marker effect estimation; Figure 1 (.zip, 170 KB)
- File S2 - R code for data simulation and effect estimation; R code for plotting estimated marker effects of the simulated data; Figure 2; Estimated marker effects used for plotting in fig-2-plot.R (.zip, 7 MB)
- File S3 - R code for converting the data of Crossa et al. (2010) for SelectionTools; R code for converting the data of Pérez-Rodríguez et al. (2012) for SelectionTools; R code for computing times of marker effect estimation in the data set of Crossa et al. (2010); R code for computing times of marker effect estimation in the sugar beet data set; R code for computing times of marker effect estimation in the data set of Pérez-Rodríguez et al. (2012); R code for computing times of marker effect estimation in the simulated data set; and genotypic and phenotypic data of sugar beet dataset (.zip, 234 KB)
- File S4 - R code for converting the data of Crossa et al. (2010) for SelectionTools; R code for converting the data of Pérez-Rodríguez et al. (2012) for SelectionTools; R code for cross validation in the data set of Crossa et al. (2010); R code for cross validation in the data set of the sugar beet data set; R code for cross validation in the data set of Pérez-Rodríguez et al. (2012); genotypic and phenotypic data of sugar beet dataset, trait SC; and genotypic and phenotypic data of sugar beet dataset, trait ML (.zip, 459 KB)
- Figure S5 - Marker effects (blue circles) estimated with different GWP approaches in the simulated data set plotted against marker locations [M] for the first chromosome. (.zip, 72 KB)
