# Supplementary figures and images for: Heteroscedastic Ridge Regression Approaches for Genome-Wide Prediction With a Focus on Computational Efficiency and Accurate Effect Estimation
Source: G3 (Bethesda). 2014 Jan 21;4(3):539–46. doi: 10.1534/g3.113.010025 (PMC3962491; doi:10.1534/g3.113.010025)

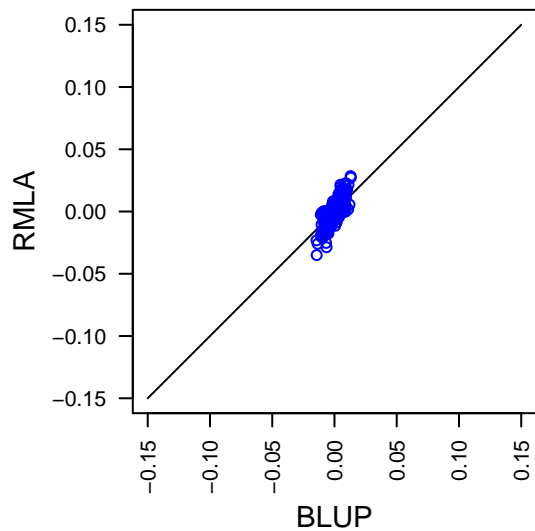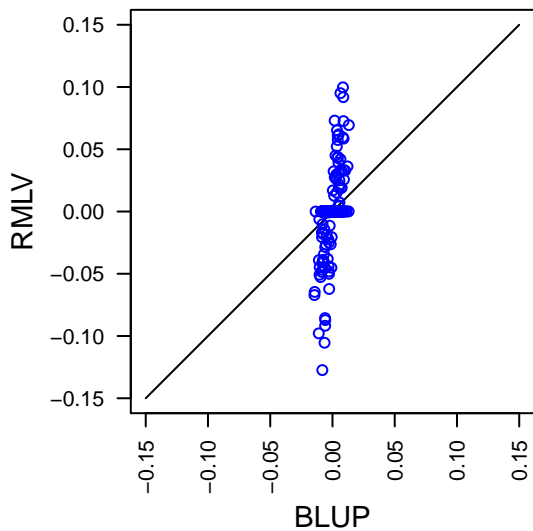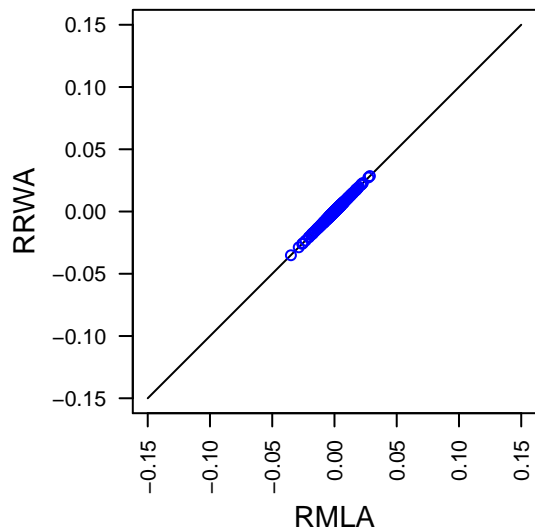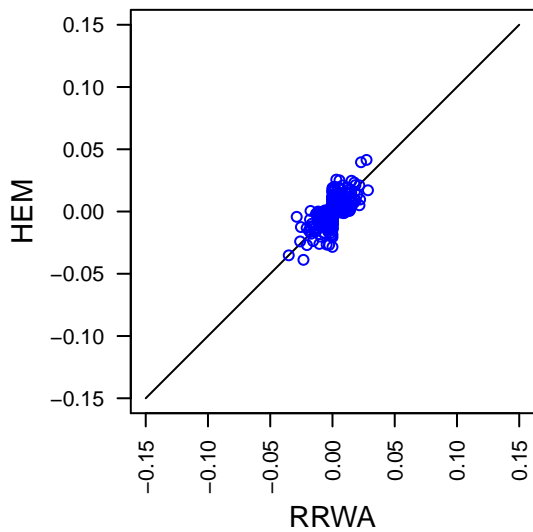

Supplement: Supporting Information [file supp_g3.113.010025_FileS1.zip › FileS1/fig-1.pdf]

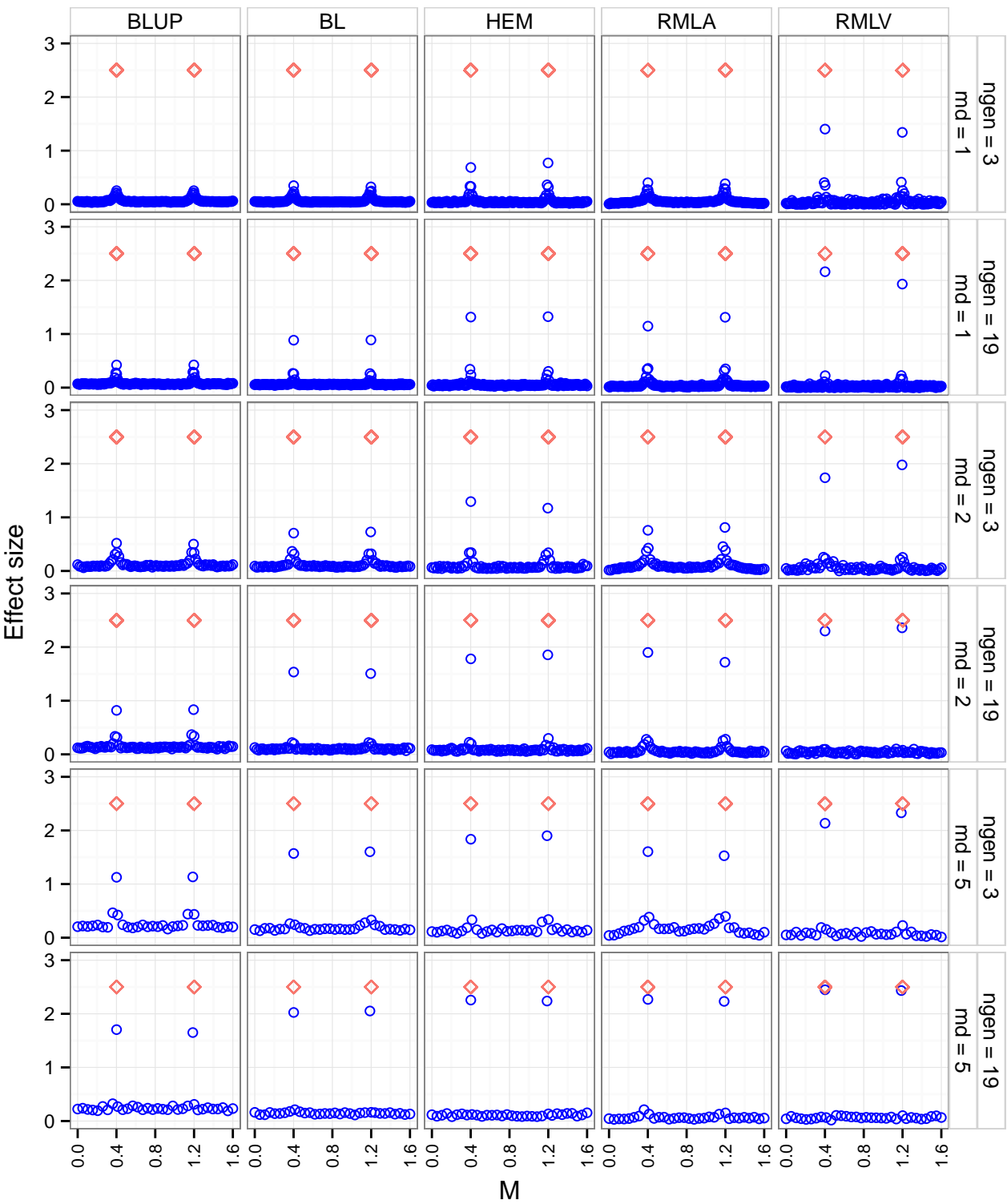

Supplement: Supporting Information [file supp_g3.113.010025_FileS2.zip › FileS2/fig-2.pdf]
