# Supplementary material for: Heteroscedastic Ridge Regression Approaches for Genome-Wide Prediction With a Focus on Computational Efficiency and Accurate Effect Estimation
Source: G3 (Bethesda). 2014 Jan 21;4(3):539–46. doi: 10.1534/g3.113.010025 (PMC3962491; doi:10.1534/g3.113.010025)
Supplement: Supporting Information [file supp_g3.113.010025_FileS5.zip › FileS5/FigureS5.pdf]

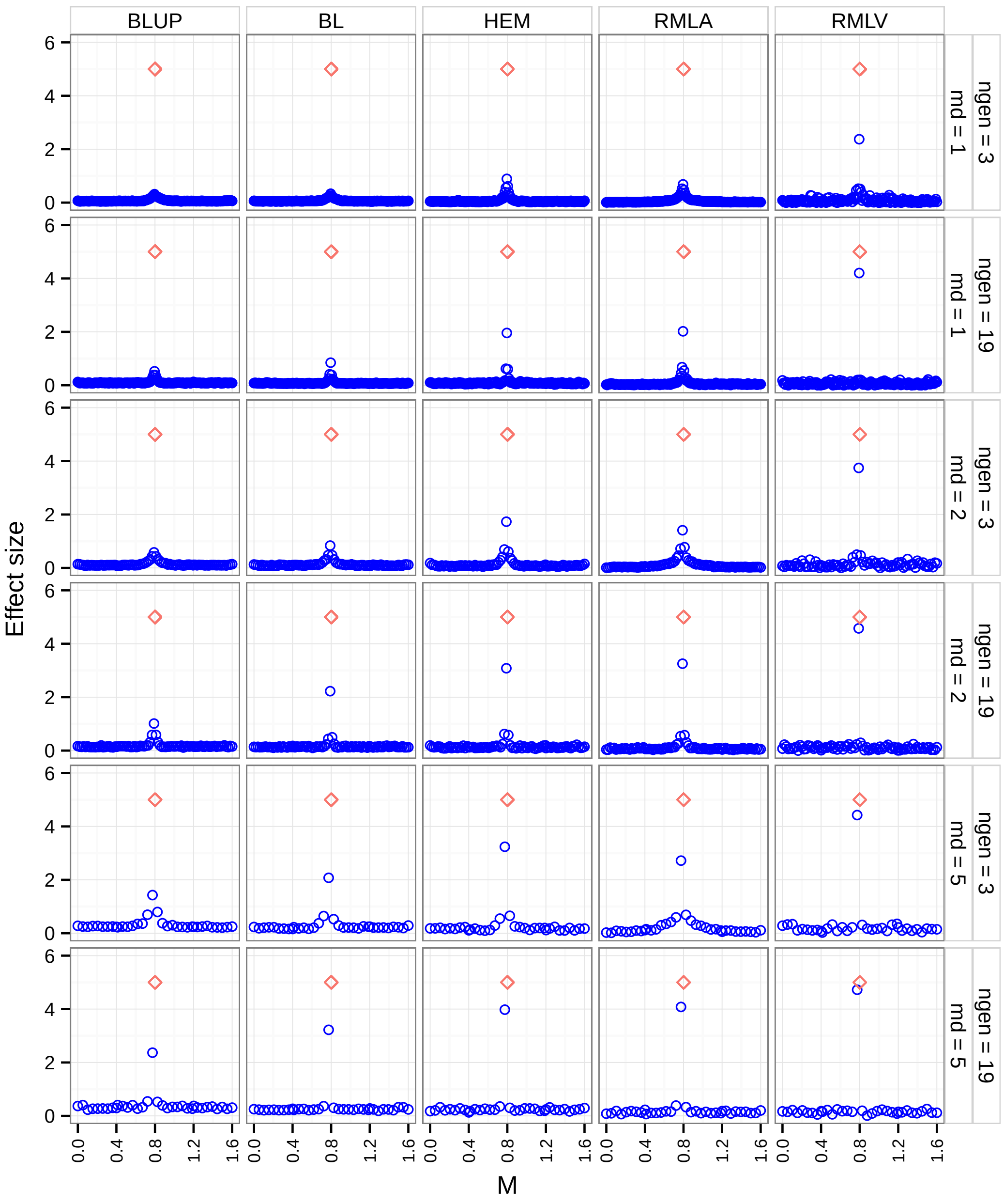

Fig. S 5 Marker effects (blue circles) estimated with different GWP approaches in the simulated data set plotted against marker locations [M] for the first chromosome. The positions of the simulated QTL are symbolized by open red diamonds, ngen is the number of random intermating generations, md is the marker distance [cM] of two adjacent markers. For the simulation we assumed that one gene affected the trait on each chromosome, they were 0.801 cM distant from the teleomere. Each gene had a postitve effect of 5 on the trait. The heritability of the trait was $h^2 = 0.5$.
